# Impact of gastroesophageal reflux disease on daily life: the Systematic Investigation of Gastrointestinal Diseases in China (SILC) epidemiological study

**DOI:** 10.1186/1477-7525-8-128

**Published:** 2010-11-10

**Authors:** Rui Wang, Duowu Zou, Xiuqiang Ma, Yanfang Zhao, Xiaoyan Yan, Hong Yan, Jiqian Fang, Ping Yin, Xiaoping Kang, Qiang Li, John Dent, Joseph J Sung, Katarina Halling, Saga Johansson, Wenbin Liu, Jia He

**Affiliations:** 1 Second Military Medical University, Shanghai, China; 2 Xi'an Jiao Tong University, Xi'an, China; 3 Zhongshan Medical University, Guangzhou, China; 4 Huazhong Science and Technology University, Wuhan, China; 5 Peking University, Beijing, China; 6 Royal Adelaide Hospital, Adelaide, SA, Australia; 7 Chinese University of Hong Kong, Hong Kong, China; 8 AstraZeneca R&D, Mölndal, Sweden; 9 AstraZeneca Pharmaceutical Company Limited, Shanghai, China

## Abstract

**Background:**

Gastroesophageal reflux disease imposes a significant burden of illness in Western populations. However, data on the impact of reflux symptoms on daily life in Asian populations are scarce. The current study aimed to evaluate the impact of GERD (defined on the basis of symptoms) on health-related quality-of-life (HRQoL) in individuals from five regions in China, as part of the Systematic Investigation of Gastrointestinal Diseases in China (SILC) study.

**Methods:**

In total, 18 000 residents were randomly selected from across five regions of China and asked to complete a general information questionnaire and a Chinese version of the Reflux Disease Questionnaire (RDQ). A randomly selected subsample of one-fifth of subjects (20% from each region) completed Chinese versions of the 36-item self-administered (SF-36) questionnaire and Epworth Sleepiness Scale (ESS) questionnaire. Reflux symptoms were defined as the presence of heartburn and/or regurgitation. Symptom-defined GERD was diagnosed as mild heartburn and/or regurgitation ≥2 days per week, or moderate/severe heartburn and/or regurgitation ≥1 day a week, based on the Montreal Definition of GERD for population-based studies.

**Results:**

The response rate was 89.4% for the total sample (16 091/18 000), and for the 20% subsample (3219/3600). Meaningful impairment was observed in all 8 SF-36 dimensions in participants with symptom-defined GERD, in 7 of the 8 SF-36 dimensions in participants with troublesome reflux symptoms, and in 6 of the 8 SF-36 dimensions in participants with reflux symptoms below the threshold for symptom-defined GERD. Meaningful daytime sleepiness was also observed in each of these groups. The proportion of individuals reporting troublesome symptoms increased as reflux symptom frequency and severity approached the threshold for symptom-defined GERD, and this was associated with concomitant decreases in all HRQoL measures. Troublesome symptoms were reported by 68.2% (75/110) of individuals with symptom-defined GERD.

**Conclusions:**

GERD diagnosed using symptom/frequency criteria (recommended for population-based studies), or based on troublesome reflux symptoms (recommended for the clinic), is associated with significantly impaired HRQoL in Chinese individuals. However, patient groups identified using these definitions do not overlap completely, suggesting that they capture slightly different, though clinically relevant, GERD populations.

## Background

Gastroesophageal reflux disease (GERD) has been shown to impose a significant and meaningful burden of illness on individuals in several Western population-based studies that have used validated questionnaires [1-5]. Indeed, even mild reflux symptoms have been shown to impair health-related quality-of-life (HRQoL) [1]. The burden associated with reflux symptoms encompasses impaired physical activity, psychosocial well-being and daily functioning, as well as reduced vitality and disturbed sleep [1,3,4]. Impaired HRQoL associated with reflux symptoms leads to reduced work productivity, which incurs substantial economic costs [6,7].

Data on the impact of reflux symptoms on HRQoL in Asian populations are scarce, and cannot be inferred using data from Western countries because cultural differences are likely to modulate the impact of reflux symptoms on daily living. One epidemiological study of GERD in Guangzhou, South China, evaluated HRQoL impairment in individuals with GERD using a Chinese version of the 36-item Short-Form Health Survey (SF-36) [8]. Compared with controls, individuals with GERD in this study experienced considerable impairment in HRQoL, particularly in the bodily pain, role limitation-physical and role limitation-emotional dimensions. We have previously reported a pilot population-based study (n = 1034) of the burden of GERD in Shanghai, China [9] where GERD was associated with meaningfully reduced HRQoL, particularly with regard to the bodily pain, general health and role limitation-physical dimensions. The current study aimed to evaluate the impact of GERD (defined on the basis of symptoms) on HRQoL in individuals from five regions in China, and was part of the Systematic Investigation of Gastrointestinal Diseases in China (SILC) study.

## Methods

### Setting, sampling and study design

The populations of Shanghai, Beijing, Xi'an, Wuhan and Guangzhou were selected for sampling in this study. These regions are major population centres of eastern, northern, western, central and southern China. The survey was conducted between April 2007 and January 2008.

As described in detail elsewhere [10], 18 000 residents of China aged 18-80 years (n = 3600 from each study region) were randomly selected using a stratified, multi-stage sampling methodology. Urban and rural populations were sampled in equal numbers within each study region. Residents were randomly selected from urban areas or villages in proportion to the age and gender distributions for each region, according to population census statistics published by the government. Three attempts were made to contact a resident before he or she was considered to be a non-responder.

All respondents completed a general information questionnaire and a Chinese version of the Reflux Disease Questionnaire (RDQ). A randomly selected subsample of one-fifth of subjects (20% from each region) also completed Chinese versions of the SF-36 and Epworth Sleepiness Scale (ESS) questionnaire, and underwent a physical examination.

The study supervisors who developed the surveys and organized their administration were graduates from the Department of Health Statistics, Second Military Medical University in Shanghai, who received training from gastrointestinal specialists and epidemiologists in Shanghai. The study supervisors provided standardized training for the survey facilitators, who were local university graduates or social workers from the sampled sites. Questionnaires were self-administered in local residential committee offices or in residents' own homes, and the survey facilitators were available to explain any questions that were unclear.

Informed consent was obtained from all subjects, who were free to discontinue their participation in the study at any time. The study was approved by the Ethics Committee of the Second Military Medical University, Shanghai, China.

### Questionnaires

#### General information questionnaire

The general information questionnaire was used to collect self-reported data on age, height, weight, sex, marital status, education, income, occupation, lifestyle habits, psychological stress, family history of gastrointestinal diseases, current health status and medical history (self-reported physician diagnoses and related treatments) [11].

#### RDQ

The RDQ is a self-report questionnaire that assesses the presence of heartburn/chest pain, regurgitation and epigastric pain/burning over the previous 4-weeks using a set of six items (two items per symptom) in relation to both symptom frequency and severity (12 items total). Each item is scored on a 6-point Likert scale for frequency (0, no symptoms; 1, symptoms <1 day a week; 2, symptoms 1 day a week; 3, symptoms 2-3 days a week; 4, symptoms 4-6 days a week; 5, daily symptoms) and severity (0, no symptoms; 1, very mild symptoms; 2, mild symptoms; 3, moderate symptoms; 4, moderately severe symptoms; 5, severe symptoms). The Chinese RDQ used in the present study had a 1-week recall period which had credible reliability and construct validity when used in the pilot study [11].

The RDQ has recently been documented as a diagnostic tool for GERD in patients consulting with upper gastrointestinal symptoms in primary care [12] and as a means of monitoring treatment response [13,14].

In the current study, an additional question was added to the Chinese RDQ asking whether the symptoms were troublesome or not. This question was included because the Montreal Definition of GERD states that, in the clinic, GERD should be considered present when the reflux of stomach contents causes symptoms that are troublesome [15]. However, the format of the questionnaire did not allow participants to specify which of the RDQ symptoms (epigastric, heartburn, regurgitation) they found troublesome. Therefore, participants in whom troublesome symptoms were definitely caused by reflux (i.e. heartburn and/or regurgitation) could only be identified by excluding all participants with epigastric symptoms.

#### SF-36

The SF-36 is a generic 36-item self-administered questionnaire [16] that measures HRQoL according to eight dimensions: physical functioning, role limitation-physical, bodily pain, general health, vitality, social functioning, role limitation-emotional and mental health. Raw scores are transformed into a value falling on a 0-100 scale, with lower scores denoting impaired HRQoL. The reliability and validity of the SF-36 are well documented in a range of language versions, including Chinese [17-19].

#### ESS questionnaire

The ESS is an eight-item, self-administered questionnaire used to score the likelihood of daytime sleepiness in various situations [20]. Responses are scored on a 4-point Likert scale (3, high risk of dozing; 0, no risk of dozing). Item scores are summed to produce a final score ranging from 0 to 24. The reliability and internal consistency of the ESS has been demonstrated in Australia in English [21] and in Hong Kong in Chinese [22].

### Definitions

Reflux symptoms were defined as the presence of heartburn and/or regurgitation. Heartburn was assessed using the 'burning behind the breastbone' and 'pain behind the breastbone' items of the RDQ, and regurgitation was assessed using the 'acid taste in your mouth' and 'unpleasant movement of materials upwards from the stomach' items of the RDQ. Using the Montreal Definition of GERD for population-based studies, symptom-defined GERD in the SILC study was diagnosed based on the presence of mild symptoms of heartburn and/or regurgitation occurring on at least 2 days per week (RDQ item severity score of 2 for a frequency score ≥3), or moderate/severe symptoms of heartburn and/or regurgitation occurring on at least 1 day per week (RDQ item severity score ≥3 for a frequency score ≥2) [15].

A meaningful impairment in HRQoL was defined as a significant (*p *< 0.05) decrease of ≥5 points in a 100-point SF-36 dimension [16]. Meaningful daytime sleepiness was defined as an ESS score of >12 (a score of 10-12 was borderline and <10 was normal) [20].

### Data collection and statistical analysis

Data were collected and validated as previously described [10]. If 50% or more of items were completed in one dimension of the SF-36 and ESS questionnaire, the mean value of the completed items in that dimension was used to impute any missing values. If more than 50% of the items were missing, the score was excluded from the analysis (as recommended in the SF-36 manual). The SAS 9.1.3 (SAS Institute, Cary, NC, USA) program was used to analyse the data. We determined the statistical significance of continuous variables using *t-*tests or analysis of variance (ANOVA). ESS scores were compared between groups using the Cochran-Mantel-Haenszel test or Wilcoxon rank sum test. All of the above hypothesis tests were two-sided, and a two-tailed *p*-value of 0.05 or less was considered to indicate statistical significance.

## Results

### Response rate and sample characteristics

The response rate was 89.4% (16 091/18 000), and 16 078 responses were suitable for analysis. Demographic characteristics of the whole study population are described fully elsewhere [10]. The response rate in the 20% subsample taken across the five SILC study sites was 89.4% (3219/3600), with sufficient SF-36 data obtained from 89.3% (3214/3600) of individuals and sufficient ESS data obtained from 88.9% (3200/3600) of individuals. Where there were not enough data available for inclusion this was due to logistical errors and/or insufficient completion of the questionnaire. Characteristics of the 20% subsample were very similar to those reported for the study population as a whole (Table 1), indicating that this is a representative subgroup of the entire SILC study population. The prevalence of symptom-defined GERD in the subsample (3.4%) was very similar to that in the total SILC study population (3.1%).

**Table 1:** Demographic and baseline characteristics of the 20% subsample that completed the SF-36 and ESS questionnaire, compared with the full study population.

	Entire SILC study population N = 16 078	20% subsample N = 3214
	
	n (%)	n (%)
**Region**		
Urban	8072 (50.2)	1585 (49.3)
Rural	8006 (49.8)	1629 (50.7)
**Sex**		
Female	8390 (52.2)	1678 (52.2)
Male	7688 (47.8)	1536 (47.8)
**Age (years)**		
18-29	3680 (22.9)	749 (23.3)
30-39	3675 (22.9)	736 (22.9)
40-49	3812 (23.7)	757 (23.6)
50-59	2468 (15.4)	486 (15.1)
60-69	1503 (9.3)	309 (9.6)
70-80	940 (5.8)	177 (5.5)
**Smokers**	4431 (27.6)	859 (26.7)
**Alcohol consumption (any)**	3262 (20.3)	684 (21.3)
**Body mass index (kg/m**^**2**^**; mean ± SD)**	22.6 ± 3.3	22.6 ± 3.4

ESS, Epworth Sleepiness Scale; SD, standard deviation; SF-36, 36-item Short-Form Health Survey; SILC, Systematic Investigation of Gastrointestinal Diseases in China.

### Symptom-defined GERD and HRQoL

**Figure 1:**
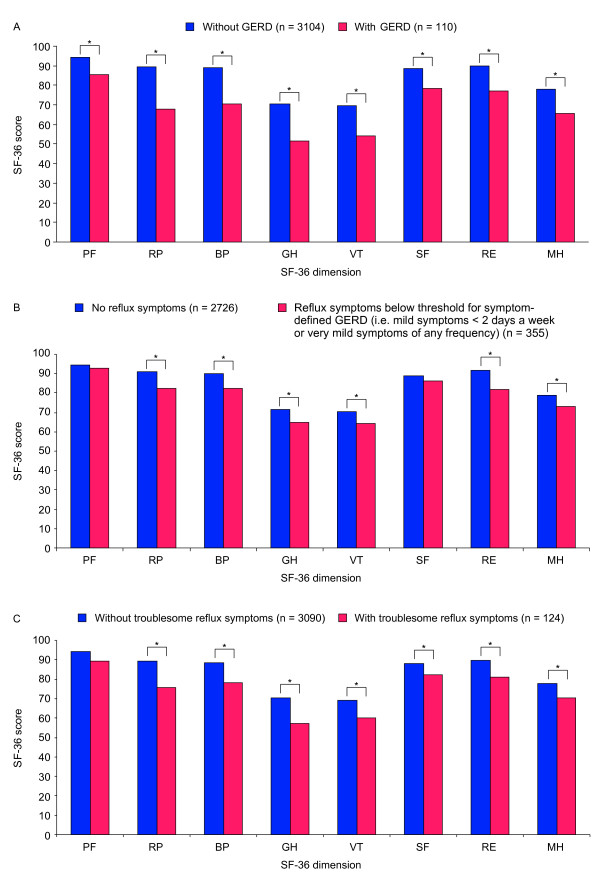
**Dimension scores of the 36-item Short-Form Health Survey (SF-36) in A) participants with and without symptom-defined gastroesophageal reflux disease (GERD), B) participants with and without reflux symptoms below the threshold for symptom-defined GERD and C) patients with and without troublesome reflux symptoms**. PF, physical functioning; RP, role limitation-physical; BP, bodily pain; GH, general health; VT, vitality; SF, social functioning; RE, role limitation-emotional; MH, mental health. *Meaningful impairment (statistically significant [*p *< 0.05] decrease of ≥5 points).

In the subsample, subjects with symptom-defined GERD had meaningfully impaired HRQoL in all eight SF-36 dimensions compared with those without (Figure 1A). The mean reduction in HRQoL in individuals with symptom-defined GERD was greater for the role limitation-physical (-21.8 points), general health (-19.1 points), bodily pain (-18.3 points) and vitality (-15.3 points) dimensions than for the other four dimensions (range, -8.8 to -13.1 points). However, the impact of symptom-defined GERD was not uniform across the five sample regions. Specifically, only the bodily pain and general health dimensions were meaningfully impaired by symptom-defined GERD in the Guangzhou region (-12.4 points and -12.6 points; *p *< 0.01), while social functioning was not meaningfully impaired in the Beijing region (-5.9 points; *p *= 0.11), and physical functioning (-2.8 points; *p *= 0.30) and role limitation-emotional (-4.2 points; *p *= 0.58) dimensions were not meaningfully impaired in the Xian region.

**Figure 2:**
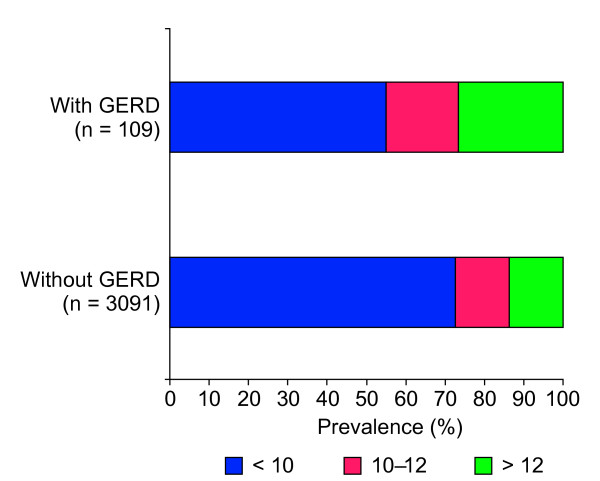
The distribution of Epworth Sleepiness Scale (ESS) scores (clinically meaningful [>12], borderline clinically meaningful B1010B1111B1212 and non-clinically meaningful [<10]) among patients with and without symptom-defined gastroesophageal reflux disease (GERD).

Meaningful daytime sleepiness was significantly more prevalent among participants with symptom-defined GERD than among those without (26.6% versus 13.8%; *p *< 0.0001) (Figure 2).

### Reflux symptom frequency and severity, and HRQoL

**Figure 3:**
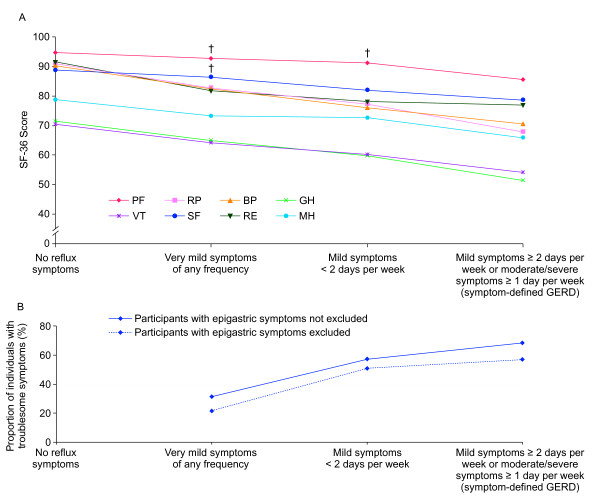
**The relationship between reflux symptom frequency/severity and 36-item Short-Form Health Survey (SF-36) dimension scores**. PF: physical functioning; RP: role limitation-physical; BP: bodily pain; GH: general health; VT: vitality; SF: social functioning; RE: role limitation-emotional; MH: mental health. † = not meaningfully impaired compared to participants with no reflux symptoms.

Participants with reflux symptoms below the threshold for symptom-defined GERD (i.e. mild reflux symptoms <2 days a week or very mild reflux symptoms of any frequency) had meaningfully impaired HRQoL in all SF-36 dimensions except role physical functioning and social functioning, compared with participants who had no reflux symptoms (Figure 1B). A clear trend was observed towards greater impairment in all SF-36 dimensions as reflux symptom frequency and severity approached the threshold for symptom-defined GERD (Figure 3A). Participants with very mild reflux symptoms of any frequency had meaningful impairment in six of the eight SF-36 dimensions (physical functioning and social functioning were not meaningfully impaired) compared with those without any reflux symptoms, while participants with mild reflux symptoms <2 days per week had meaningful impairment in seven of the eight SF-36 dimensions (physical functioning was not meaningfully impaired).

Meaningful daytime sleepiness was significantly more prevalent among patients with reflux symptoms below the threshold for symptom-defined GERD than among those without reflux symptoms (23.7% versus 12.4%; *p *< 0.0001).

### Troublesome symptoms and HRQoL

Meaningfully impaired HRQoL was observed in seven of the eight SF-36 dimensions (physical functioning was not meaningfully impaired) among individuals in whom troublesome symptoms were definitely due to reflux (i.e. patients reporting troublesome symptoms that had heartburn and/or regurgitation but no epigastric symptoms) and the pattern of impairment was similar to that observed in patients with symptom-defined GERD (Figure 1C versus Figure 1A). Meaningful daytime sleepiness was also significantly more prevalent among individuals with troublesome reflux symptoms (22.1% versus 13.9%; *p *< 0.05), than among those without.

The proportion of subjects with symptom-defined GERD who reported that their symptoms were troublesome was 68.2% (75/110) (Figure 3B). When participants with epigastric symptoms were excluded so that the term troublesome could be attributed solely to reflux, this percentage dropped to 56.9% (37/65). Of those participants who had reflux symptoms below the threshold for symptom-defined GERD, 40.0% (142/355) reported troublesome symptoms. This percentage dropped to 29.9% (76/254) when participants with epigastric symptoms were excluded.

The proportion of participants reporting the presence of troublesome symptoms increased as reflux symptom frequency and severity approached the threshold for symptom-defined GERD (Figure 3B). This trend remained when participants with epigastric symptoms were excluded, although the percentage of participants reporting their symptoms as troublesome was lower overall.

## Discussion

### Results in context

In this 20% subsample of the large population-based SILC study, conducted in five regions of China, GERD (defined as mild symptoms of heartburn and/or regurgitation occurring on at least 2 days per week, or moderate/severe symptoms of heartburn and/or regurgitation occurring on at least 1 day a week) was associated with meaningful impairment in HRQoL. Meaningful impairment in HRQoL was also observed among participants with reflux symptoms below the threshold for symptom-defined GERD (mild reflux symptoms <2 days a week or very mild reflux symptoms of any frequency), even when only very mild reflux symptoms were present.

The finding of significantly reduced SF-36 dimension scores and impaired sleep among participants with symptom-defined GERD compared with those without is consistent with the results of the Shanghai pilot study [9], although the pilot study did not find meaningfully impaired social functioning in individuals with reflux symptoms. In addition, the pattern of impairment observed across the different SF-36 dimensions in the current study is similar to the pattern observed in the pilot study and in a previous population-based study conducted in southern China [8]. It is possible that the impact of symptom-defined GERD on SF-36 dimensions such as role limitation-emotional, social functioning, and mental health, are secondary to its effects on physical dimensions such as role limitation-physical, bodily pain and general health, which are likely to occur as a direct consequence of the painful nature of this disease. This idea is supported by the greater impact of symptom-defined GERD on the latter SF-36 dimensions compared with the former, and by previous observations of reduced emotional impairment in patients with GERD after acid-suppressive therapy [2]. Furthermore, even in the Guangzhou region, where symptom-defined GERD seemed to have a minimal impact on health-related quality of life, measures of bodily pain and general health were still meaningfully impaired by this disease. It is possible that cultural variation may modulate the impact that physical impairments caused by symptom-defined GERD can have on other aspects of daily activity. However, the complexity of the differences between these regions makes it difficult to provide a specific explanation for this observation.

Studies within mainland China indicate a link between the presence of reflux symptoms and stress [23], and between reflux symptoms and specific mental disorders such as anxiety and depression [24]. Although prone to selection bias caused by low response rates (46-63%), similar findings have also been observed in individuals in Hong Kong [25-27]. These findings are consistent with impaired mental health scores in individuals with symptom-defined GERD in the current study, and in other studies in which the SF-36 has been used [8,9].

Associations between reflux symptoms and impaired HRQoL have also been reported in individuals from Germany, Austria, Switzerland, Sweden (all using the SF-36) [3,28] and North America (using the 8-item Short-Form Health Survey) [7]. In addition, severe reflux symptoms have been associated with anxiety and depression in Norwegian populations [29]. In the Swedish general population, individuals with reflux symptoms have been shown to have impaired psychological well-being (assessed by the Psychological General Well-Being index) [1], and similar results have been observed in populations from North America, Europe and Japan [30,31]. Interestingly, Wiklund *et al*. (2006) found that mild reflux symptoms were also associated with impaired psychological well-being, which is consistent with impaired mental health scores observed in participants with mild and very mild reflux symptoms in the current study.

### Clinical implications

The Montreal Definition states that GERD is present when reflux causes troublesome symptoms that adversely affect a patient's well-being, and the symptom frequency and severity threshold used in the current study are recommended by the Montreal Definition for identifying such individuals in population-based studies [15]. The validity of the Montreal Definition of GERD for population-based studies is supported in our study by the observation of meaningfully impaired HRQoL in individuals with reflux symptoms meeting this definition. However, in the clinical setting, the Montreal Definition recommends that patients should determine if their reflux symptoms are troublesome. In our study, at least 30% of Chinese participants with heartburn and/or regurgitation below the threshold for symptom-defined GERD (Montreal definition for population-based studies) found these symptoms troublesome and had meaningfully impaired HRQoL, and would therefore be diagnosed with GERD in the clinic based on the Montreal Definition. Conversely, over 30% of individuals who met the reflux symptom frequency/severity threshold for symptom-defined GERD did not describe their symptoms as troublesome and should not be diagnosed with GERD. It therefore appears that, despite substantial overlap, GERD populations captured using the Montreal Definitions of GERD may vary slightly depending on the version used, although both versions capture individuals with impaired HRQoL.

### Strengths and limitations

The current study is the largest population-based epidemiological investigation of GERD ever conducted in China, and spans five major population centres that capture both rural and urban regions. Further strengths of this study include the use of validated questionnaires and a validated survey methodology [11], and the high response rate, which minimized the potential for responder bias. Moreover, this is the first ever study to assess the impact of symptom-defined GERD, as diagnosed using the Montreal Definition, on measures of HRQoL.

One of the limitations of this study was the inability to distinguish (due to the format of the questionnaire) which of the three symptom groups (heartburn, regurgitation or epigastric) participants found troublesome. The only way to identify participants in whom troublesome symptoms were definitely caused by heartburn and/or regurgitation was to exclude participants with any epigastric symptoms. However, this blunt approach would have also excluded some individuals whose troublesome symptoms were in fact caused by heartburn and/or regurgitation, and the size of this group was therefore probably underestimated.

Another potential limitation of this study is that impaired HRQoL in patients with symptom-defined GERD, as measured using the SF-36 and ESS questionnaire, may be related to factors associated with symptom-defined GERD (e.g. other upper gastrointestinal diseases, anxiety, depression), rather than to the presence of troublesome reflux symptoms. Arguing against this possibility is the observation that the proportion of individuals who reported their symptoms as troublesome increased as reflux symptom frequency and severity approached the threshold for symptom-defined GERD, and that this was associated with concomitant decreases in all HRQoL measures. In addition, participants who described their reflux symptoms as troublesome had patterns of impaired HRQoL that were similar to those observed in patients who met the criteria for symptom-defined GERD. These data suggest that impaired HRQoL in individuals with symptom-defined GERD, and in patients with reflux symptoms below the threshold for symptom-defined GERD, was related to the presence of troublesome reflux symptoms. However, as with any cross-sectional study, the direction of this relationship could not be assessed.

### Future work

Further research is needed to assess how reflux symptom type, frequency and severity influences treatment outcomes and consultation behaviour in Chinese individuals. In addition, correlating therapy-induced improvements in reflux symptoms with improved HRQoL would help to clarify the directionality of the relationship between reflux symptoms and impaired HRQoL in this population. Finally, population-based studies using the Montreal Definition of GERD also need to be conducted in Western countries to allow a direct comparison of the impact of symptom-defined GERD on daily life between these culturally distinct populations.

## Conclusion

GERD diagnosed based on symptom/frequency criteria (recommended by the Montreal Definition for use in population-based studies), or based on the presence of troublesome reflux symptoms (recommended by the Montreal Definition for use in the clinic), is associated with significantly impaired HRQoL in Chinese individuals. Groups identified using these definitions do not overlap completely, suggesting that they capture slightly different, though clinically relevant, GERD populations.

## Competing interests

RW, DZ, XM, YZ, XY, HY, JF, PY, XK and QL declare that they have no competing interests. JS has served as a speaker, a consultant and an advisory board member for AstraZeneca, and has received research funding from AstraZeneca. SJ is an employee of AstraZeneca. KH was an employee of AstraZeneca at the time the study was conducted and is now employed by PRO Consulting. WL was an employee of AstraZeneca at the time the study was conducted, and is now employed by Sanofi-Aventis (China). JH has served as the Director of the Department of Health Statistics, Second Military Medical University and has received research funding from AstraZeneca. JD has served as a speaker, a consultant and an advisory board member for AstraZeneca, and has received research funding from AstraZeneca.

## Authors' contributions

JH, XM, YZ, RW, XY, JD, JS, DZ, SJ, KH, WL and ZL made substantial contributions to the conception and design of the study. JH, XM, YZ, RW, XY, HY, PY, XK, JF, QL and WL participated in data collection. JH, XM, YZ, RW, XY, JD, JS, DZ and SJ analyzed and interpreted the data. All authors have been involved in critically revising the manuscript for intellectual content, and have given final approval of the version to be published.
